# How to Manage Otorrhea

**Published:** 1907-04

**Authors:** 


### ABSTRACTS.

### How to Manage Otorrhea.

F. E. BURGEVIN, of Spiro, I. T., says (*Kansas City Medical Record,* July, 1906,) that otorrhea was formerly his bete noire. He employed the treatment of Pomeroy and others, which he learned when he was serving at the Manhattan Eye and Ear Hospital in 1890. This consisted of syringing, insufflations of powdered boric acid and the like, sometimes with benefit but rarely with cure. His present method, simple and effectual, consists of first filling the ear with a warm solution of some good peroxide of hydrogen, beginning with a 25% solution, increasing the strength every day until the pure drug is used. This of course is done only once a day. Hydrozone, which is twice as strong, he uses instead from motives of economy. After cleansing the ear thoroughly, which at first may require from twenty minutes to two hours according to the degree of foulness of the auditory canal, he then instils a few drops of warm glycozone and closes the canal securely with absorbent cotton which is allowed to remain until the next treatment.

The first cleansing should be thorough, the peroxide being repeatedly instilled until all foaming ceases. Sometimes three or four treatments will become necessary to cleanse the ear thoroughly, especially so if the lumen is occluded by swelling or inspissated discharge. Thenceforth the daily treatment need not consume more than ten to twenty minutes. In children who dread the procedure he does not attempt much the first time or two except to strive to win their confidence. This ordinarily is not difficult, the treatment being not at all painful and is followed by a certain sense of relief.

With the diseased ear once thoroughly cleansed, Burgevin considers his work as half done, thenceforth improvement being usually very rapid. Mastoid disease, necrosis, polyps and the like, of course require appropriate treatment; but he does not hesitate to declare that all uncomplicated cases may be cured by this treatment if it is properly carried out. He cautions to have the medicaments warm but not too hot—100 F. being about right. He also cautions to always stop up the ear with a bit of aseptic cotton, which should be just the size to securely close the meatus; if too large it will work out allowing the solution to escape, thus leaving the ear unprotected; if too small it will slip back into the canal, and thus thwart the effects of treatment.

Burgevin never syringes the ears in otorrhea regarding it as risky and useless. He usually drops a little warm solution of 5% sodium borate in the ear, to prevent a slight stinging which sometimes follows after active steps and he also dries out the canal with cotton on an applicator; this latter shauld be done very carefully through the speculum with the canal well lighted. These, however, are nonessentials being merely refinements which render the treatment a little less unpleasant. The general health, he adds, should receive attention by approprite treatment.

